# In vitro testing of explanted shunt valves in hydrocephalic patients with suspected valve malfunction

**DOI:** 10.1007/s10143-021-01564-8

**Published:** 2021-05-24

**Authors:** Christoph Bettag, Christian von der Brelie, Florian Baptist Freimann, Ulrich-Wilhelm Thomale, Veit Rohde, Ingo Fiss

**Affiliations:** 1 Department of Neurosurgery, Georg-August-Universität Göttingen, Robert-Koch-Straße 40, 37075 Göttingen, Germany; 2 Pediatric Neurosurgery, Charité Universitätsmedizin Berlin, Campus Virchow Klinikum, Berlin, Germany

**Keywords:** Hydrocephalus, Shunt valve, Ventriculo peritoneal shunt, Shunt failure

## Abstract

Diagnosis of symptomatic valve malfunction in hydrocephalic patients treated with VP-Shunt (VPS) might be difficult. Clinical symptoms such as headache or nausea are nonspecific, hence cerebrospinal fluid (CSF) over- or underdrainage can only be suspected but not proven. Knowledge concerning valve malfunction is still limited. We aim to provide data on the flow characteristics of explanted shunt valves in patients with suspected valve malfunction. An in vitro shunt laboratory setup was used to analyze the explanted valves under conditions similar to those in an implanted VPS. The differential pressure (DP) of the valve was adjusted stepwise to 20, 10, 6, and 4 cmH_2_O. The flow rate of the explanted and the regular flow rate of an identical reference valve were evaluated at the respective DPs. Twelve valves of different types (Codman CertasPlus valve *n* = 3, Miethke Shuntassistant valve *n* = 4, Codman Hakim programmable valve *n* = 3, DP component of Miethke proGAV 2.0 valve *n* = 2) from eight hydrocephalic patients (four male), in whom valve malfunction was assumed between 2016 and 2017, were replaced with a new valve. Four patients suffered from idiopathic normal pressure (iNPH), three patients from malresorptive and one patient from obstructive hydrocephalus. Post-hoc analysis revealed a significant difference (*p* < 0.001) of the flow rate between each explanted valve and their corresponding reference valve, at each DP. In all patients, significant alterations of flow rates were demonstrated, verifying a valve malfunction, which could not be objectified by the diagnostic tools used in the clinical routine. In cases with obscure clinical VPS insufficiency, valve deficiency should be considered.

## Introduction

Implantation of a ventriculo-peritoneal shunt (VPS) is the most widely used neurosurgical procedure to treat patients with hydrocephalus. However, cerebrospinal fluid (CSF) shunt failure, predominantly from infection and shunt catheter obstruction, remains a persistent problem. Reported five-year shunt survival rates for first-time shunt implantations are as low as 53.1% [9, 19, 41]. Up to 25% of patients suffering from idiopathic normal pressure hydrocephalus (iNPH) develop primary deterioration after VPS implantation within the first year [14]. Furthermore, about 20% of patients develop secondary deterioration following initial improvement after VPS implantation within two to four years [5, 14]. Gutowski et al. proposed a sophisticated algorithm for shunt management to reduce the rate of secondary deterioration from 20 to 15%. However, about 60% of these patients remained secondary non-responders, although in 27% a shunt revision was performed [14]. If revision surgery is required for reasons other than infection, neuroimaging is considered helpful in identifying the exact location of a presumed obstruction or leakage along the shunt system. Besides head computed tomography or magnetic resonance imaging (MRI) to assess ventricle size and catheter placement, shunt series are performed to search for breaks, disconnections, or catheter migration [37]. When this standard work-up fails to show structural explanation for a clinically suspected shunt malfunction, contrast-enhanced shunt series (“shuntography”) may be used as an adjunct diagnostic procedure [36]. In rare cases, however, even extended neuroimaging does not yield a cause for shunt malfunction in patients presenting with nonspecific symptoms. Then, the valve itself as the origin of the shunt malfunction may come to the fore. The aim of our study was to clarify whether in patients requiring shunt revision, without a final yield in prior diagnostics, the valve itself might contribute to the malfunction of the shunt system or not.

## Material and methods

We performed experimental in-vitro testing of explanted shunt valves. The study was approved by the local ethics committee (study number 10/4/17). Informed consent was obtained from each patient. Patients with suspected shunt malfunction underwent intensive diagnostic workup prior to surgery. In case of suspected underdrainage, low-dose CT-shunt-series as well as conventional contrast-enhanced shunt series failed to demonstrate radiological evidence of shunt failure (i.e., enlargement of ventricles compared to prior imaging or obstruction of the VPS) [13, 35, 36], while clinical symptoms were persistent despite multiple adjustments of the respective valve. After lumbar puncture, clinical symptoms improved in all of these patients so that finally valve dysfunction was suspected. In case of suspected overdrainage, an adjustable gravitational unit was implanted, and multiple valve adjustments were performed without improvement of clinical symptoms. Radiological evidence of overdrainage (e.g., subdural hygroma, slit ventricles) has been proven in none of the patients. Furthermore, in all of the non-NPH patients, lumbar puncture was performed to assess the ICP.

Revision was indicated due to missing radiological evidence of VPS failure while clinical symptoms of shunt failure persisted although intensive shunt management was performed in all of the patients.

In all cases, intraoperative testing of the shunt components was performed. CSF flow could be seen at the distal end of the proximal catheter after disconnection from the valve in all cases. Likewise, a distal outflow of the irrigation fluid was seen when a manometer was connected to the proximal inlet of the valve. The explanted valve was then examined in our experimental set-up.

### Experimental set-up

**Fig. 1:**
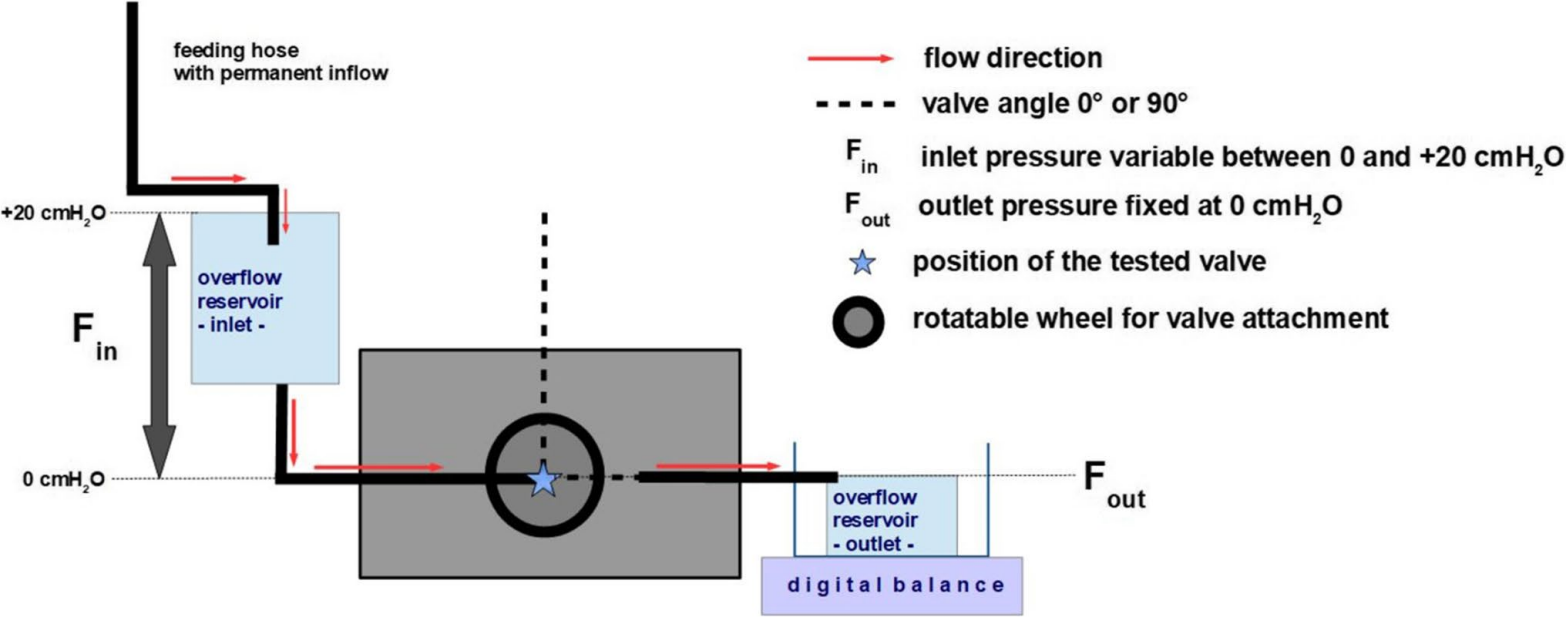
Schematic drawing of the experimental set-up.

The experimental set-up was designed to simulate the pressure environment and flow conditions similar to those in a VPS as previously described by Freimann et al. (Fig. 1) [12]. An overflow reservoir represented the intracranial compartment allowing a constant inflow pressure into the tubing. The tubing was connected with the explanted valve. The height of the inflow reservoir was adjustable in relation to the valve, simulating an inflow pressure range between + 20 and 0 cmH_2_O. The explanted valve was fixed in a horizontal and the gravity-regulated shunt assistant in a vertical position, respectively. The intraperitoneal compartment was also simulated by an overflow reservoir, which was connected downstream from the valve by silicon tubing to allow a constant outflow. The height of the reservoir was adjusted relative to the valve at a pressure of 0 cmH_2_O. Thus, the overall hydrostatic DP within the measurement set-up was adjustable from 0 to 20 cmH_2_O.

Exact alignment of the spatial position of both overflow reservoirs as well as the valve devices was ascertained using a 360° three plane leveling alignment laser (Bosch PLL 360, Robert Bosch GmbH, Stuttgart, Germany). The flow over time through the tested valve combinations was recorded by weight change at the outflow reservoir in g/min with the electronic precision scale A&D EJ-610 (A&D Company, Tokyo, Japan). The scale was connected to a personal computer via an RS232C serial interface and weight change per minute was transmitted using the software RsCom V 5.11 (A&D Company, Tokyo, Japan) [11].

### Measurement protocol

The first measurement protocol was designed to specify the flow characteristics of a new reference valve, which were adjusted at the same opening pressure of the respective explanted valve. The reference valve was a new valve of the same type as the particular explanted valve and adjusted to the same opening pressure. The adjustments of each explanted and reference valve were verified by x-ray.

The inflow pressure values simulating an intracranial pressure (ICP) of 4, 6, 10, and 20 cmH_2_O were generated by stepwise elevation of the height of the inflow reservoir above the valve. The outflow reservoir was placed at the same height as the valve.

The second measurement protocol was performed with the explanted valves in the same manner.

All measurements in the first and second protocol at each DP were carried out three times for one minute for each valve and the mean value was calculated. All measurements of the explanted valves were performed within 60 min after the end of surgery. Measurements were started after an initial irrigation for 10 min. The explanted valves were kept humid in a chamber to avoid precipitation of CFS proteins. Forced irrigation was not performed as not to change the constitution of the explanted valve. The settings of the explanted valves were verified via x-ray and the corresponding reference valve was adjusted to the identical setting. Upon inspection with a magnifying glass, no aberrant appearances of the valves were found.

### Statistical analysis

Statistical analyses were performed using SPSS 23.0 (IBM Corp., Armonk, NY, USA). Flow rates of the reference and of the explanted valves were compared with the independent *t*-test. Correlation between clinical symptoms and flow rate measurements were evaluated using Pearson correlation. *P*-values < 0.05 were considered significant. All values in the results are expressed as means ± standard deviation or median and range, respectively.

## Results

### Baseline characteristics

**Fig. 2:**
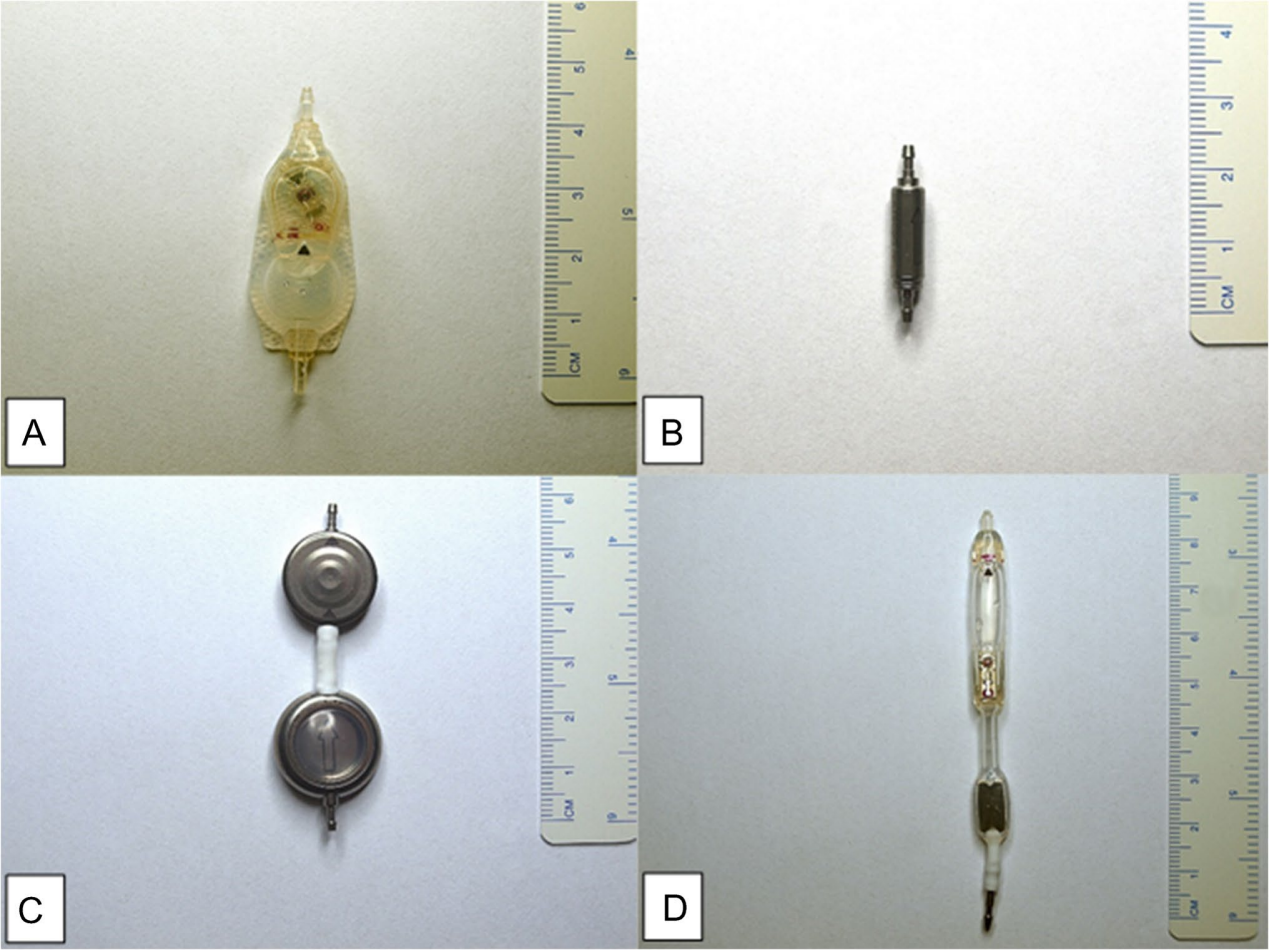
Exemplary images of the tested devices, (**a**) Codman CertasPlus valve, (**b**) Miethke gravity-regulated Shuntassistant, (**c**) the DP-component of the proGAV 2.0 valve, (**d**) Codman Hakim programmable valve

Twelve valves of different types (Codman CertasPlus valve *n* = 3, Codman Hakim programmable valve *n* = 3 (Codman Specialty Surgery, Integra LifeSciences, Plainsboro, NJ, USA), Miethke gravity-regulated Shuntassistant *n* = 4 and the DP-component of the Miethke proGAV 2.0 valve *n* = 2 (Aesculap-Miethke, Potsdam, Germany) from eight hydrocephalic patients (four males) were explanted and replaced with new valves between February and October 2017 (Fig. 2). The mean age of the patients was 63 years (range 36–79 years). Four patients had idiopathic normal pressure (iNPH), three patients posthemorrhagic and one patient obstructive hydrocephalus. The median time from initial surgery to valve explantation was 55.25 weeks (range 4–156 weeks). Development of secondary deterioration was reported in 3/8 (37.5%) of the patients after having initially improved for at least one year after VPS implantation. Primary deterioration was seen in 5/8 (62.5%) of the patients within three months after VPS implantation. All the included patients suffered from clinical symptoms of either under- or overdrainage without radiographic evidence of shunt failure. The mean time period between expressed suspicion of valve dysfunction, after radiographic exclusion of valve defects, until valve replacement was 6.0 ± 4.3 days. The median protein content of the intraoperatively taken samples of the CSF was 383 mg/l (range: 227–435 mg/l), the median cell count was 1/µl (0–4/µl) and the median lactate was 2.0 mmol/l (1.7– 2.1 mmol/l).

Over the time frame of the study, 41 VPS were implanted. In 5/41 (12.1%) of the patients, revision was required within the time frame of the study. 2/41 (4.9%) revisions were performed due to infection of the shunt system, 3/41 (7.3%) of patients underwent valve-related revisions and were subsequently included in our study. Catheter obstruction did not occur within the time frame of the study.

“In the four non-iNPH patients, lumbar puncture revealed a median opening pressure of 20 (range 12–36) cmH_2_O.”

### Clinical assessment and surgical management

In our cohort, 5/8 (62.5%) of the patients were clinically suspected to suffer from underdrainage and 3/8 (37.5%) of patients from overdrainage, respectively. The most reported clinical symptoms were gait ataxia (*n* = 6, 75.0%), followed by cognitive deterioration, position independent cephalgia and vertigo (each *n* = 5, 62.5%), urinary incontinence (*n* = 4, 50%), intention tremor (*n* = 2, 25%) and reduced vigilance status (*n* = 1, 12.5%). In average, 5.5 ± 1.4 valve adjustments were performed in each patient without significant improvement of the symptoms prior to VPS revision.

In case of suspected underdrainage, 6/12 (50%) of the implanted valves (three Codman CertasPlus, one DP component of the Miethke proGAV 2.0, one Miethke Shuntassistant and one Codman Hakim programmable valve) were replaced by Codman Hakim programmable valves in 5/8 (62.5%) of the patients. In case of suspected overdrainage, 5/12 (42%) of the implanted valves (one DP component of the Miethke proGAV 2.0, three Miethke Shuntassistants, one Codman Hakim programmable valve) were switched to Codman Certas Plus valves including a SiphonGuard as an antisiphon device (ASD) and 1/12 (8%, Codman Hakim programmable valve), was switched to a Miethke proGAV 2.0 valve combined with a fixed Shuntassistant in 3/8 (37.5%) of the patients, respectively.

### Flow rates

**Fig. 3:**
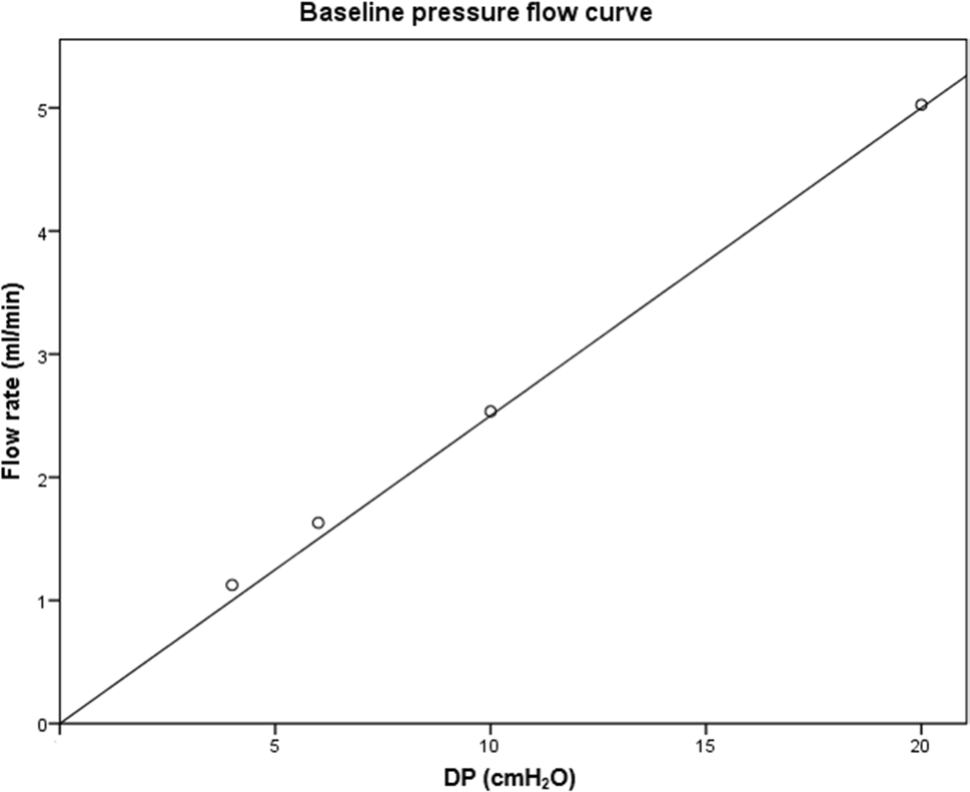
Baseline measurement showing a linear increase in flow rate with increasing differential pressure (DP) in the control measurement

The control measurement showed a linear increase in flow rate with increasing DP (Fig. 3). The mean flow rate of the reference valves for a DP of 20, 10, 6 and 4 cmH_2_O were 2.87 ± 0.01 ml/min, 1.99 ± 0.00 ml/min, 1.35 ± 0.00 ml/min and 1.05 ± 0.01 ml/min, respectively, for the Codman CertasPlus valve, 3.56 ± 0.00 ml/min, 1.73 ± 0.01 ml/min,1.04 ± 0.00 ml/min and 0.30 ± 0.01 ml/min for Miethke gravity-regulated Shuntassistant 3.84 ± 0.01 ml/min, 1.73 ± 0.01 ml/min, 1.19 ± 0.01 ml/min and 0.96 ± 0.00 ml/min for the DP-component of the Miethke proGAV 2.0 valve and 3.61 ± 0.02 ml/min, 1.55 ± 0.02 ml/min, 1.05 ± 0.00 ml/min and 0.85 ± 0.01 ml/min for the Codman Hakim programmable valve.

### Flow alterations

Measurement of the flow rates showed reduced flow in six devices (50%; three Codman CertasPlus valves, two Codman Hakim programmable valves, one DP-component of the Miethke proGAV 2.0 valve) and increased flow rates likewise in six devices (50%; four Miethke gravity-regulated Shuntassistant valves, one Codman Hakim programmable valve and one DP-component of the Miethke proGAV 2.0 valve) compared to the flow rates of the control devices.

**Fig. 4:**
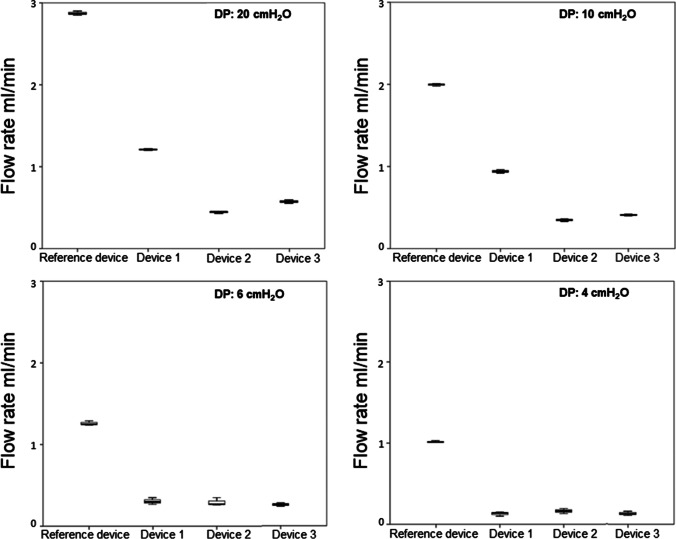
Significant flow alteration between all three explanted Codman CertasPlus devices and their corresponding reference valve at each 20, 10, 6 and 4 DP (*p* < 0.001)

**Fig. 5:**
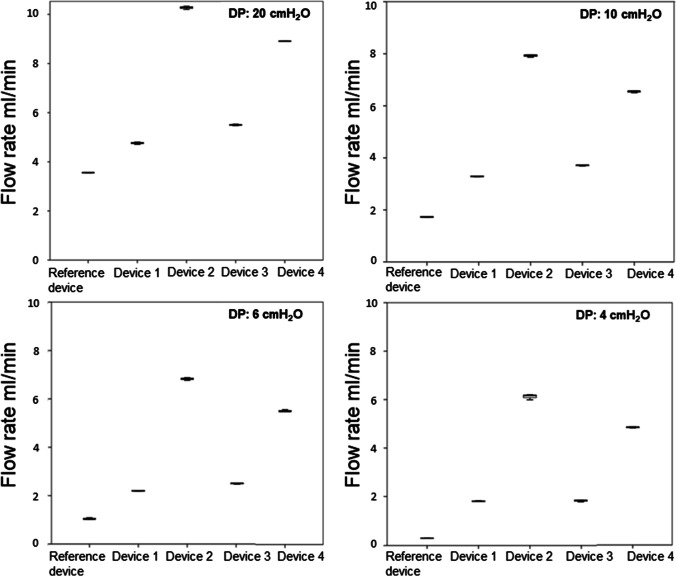
Significant flow alteration between all four explanted Miethke gravity-regulated Shuntassistant devices and their corresponding reference valve at each 20, 10, 6 and 4 DP (*p* < 0.001)

**Fig. 6:**
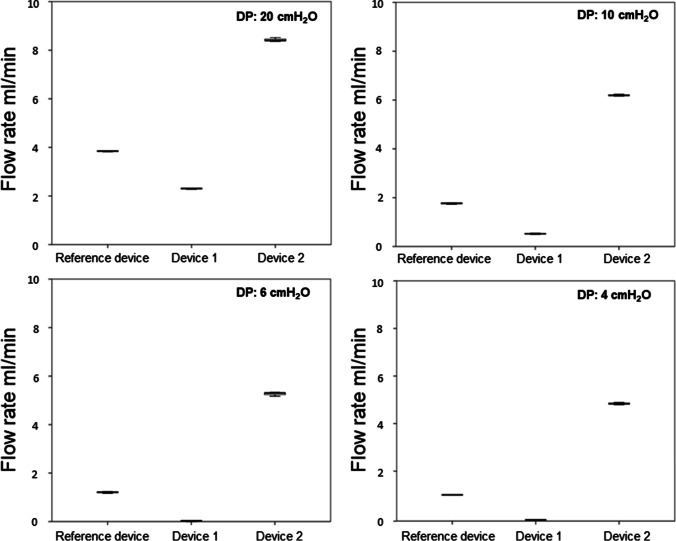
Significant flow alteration between all two explanted DP-components of the Miethke proGAV 2.0 valve and their corresponding reference valve at each 20, 10, 6 and 4 DP (*p* < 0.001)

**Fig. 7:**
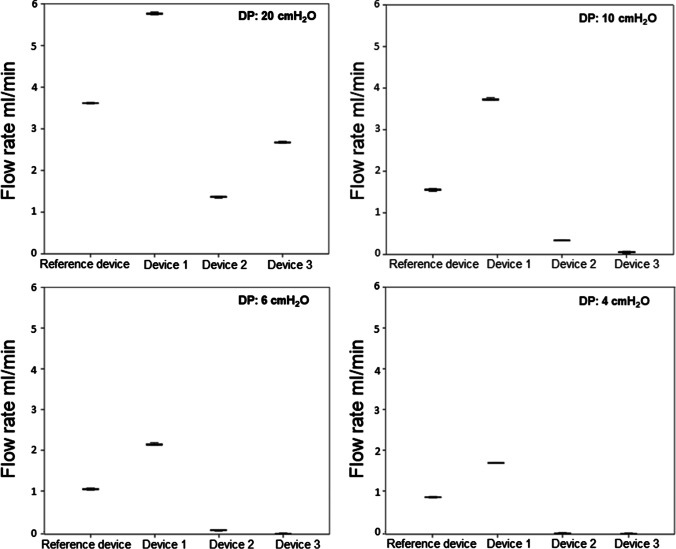
Significant flow alteration between all three explanted Codman Hakim Programmable valve and their corresponding reference valve at each 20, 10, 6 and 4 DP (*p* < 0.001)

Post-hoc analysis revealed a significant difference (*p* < 0.001) of the flow rate between each explanted shunt valves and their corresponding reference valve, at each DP (Table 1, Figs. 4, 5, 6, and 7).

**Table 1:** Flow rates and its alterations of the explanted valves compared to the respective reference valve

Shunt valve	Device no./ CSF opening pressure	DP	Mean flow rate in ml/min (SD)	Mean flow alteration in ml/min	*p*-value
Codman Certas Plus	Device # 1 0 mm H_2_O	20 10 6 4	1.21 (± 0.01) 0.94 (± 0.02) 0.38 (± 0.04) 0.24 (± 0.01)	-1.66 -1.05 -0.97 -0.81	< .0001 < .001 < .001 < .001
	Device # 2 25 mm H_2_O	20 10 6 4	0.44 (± 0.01) 0.35 (± 0.01) 0.34 (± 0.05) 0.28 (± 0.03)	-2.43 -1.64 -1.01 -0.77	< .0001 < .0001 < .001 < .001
	Device # 3 25 mm H_2_O	20 10 6 4	0.57 (± 0.02) 0.39 (± 0.01) 0.32 (± 0.01) 0.24 (± 0.02)	-2.30 -1.60 -1.03 -0.81	< .0001 < .0001 < .001 < .001
Miethke Shuntassistant	Device # 1 0/25 cm H_2_O	20 10 6 4	4.76 (± 0.04) 3.57 (± 0.03) 2.16 (± 0.01) 1.83 (± 0.02)	1.20 1.84 1.12 1.53	< .001 < .0001 < .001 < .0001
	Device # 2 0/25 cm H_2_O	20 10 6 4	10.27 (± 0.07) 7.92 (± 0.04) 6.82 (± 0.05) 6.12 (± 0.11)	6.71 6.19 5.78 5.82	< .0001 < .0001 < .0001 < .0001
	Device # 3 0/25 cm H_2_O	20 10 6 4	5.5 (± 0.03) 3.77 (± 0.02) 2.36 (± 0.01) 1.83 (± 0.03)	1.94 2.04 1.32 1.53	< .0001 < .0001 < .001 < .0001
	Device # 4 0/25 cm H_2_O	20 10 6 4	8.91 (± 0.01) 6.54 (± 0.03) 5.50 (± 0.04) 4.86 (± 0.03)	5.35 4.81 4.46 4.56	< .0001 < .0001 < .0001 < .0001
DP component of Miethke proGAV 2.0	Device # 1 1 cm H_2_O	20 10 6 4	2.30 (± 0.02) 0.52 (± 0.02) 0.02 (± 0.01) 0.00 (± 0.00)	-1.54 -1.21 -1.15 -0.96	< .0001 < .001 < .001 < .001
	Device # 2 19 cm H_2_O	20 10 6 4	8.43 (± 0.08) 6.19 (± 0.03) 5.27 (± 0.08) 4.83 (± 0.04)	4.59 4.46 4.1 3.87	< .0001 < .0001 < .0001 < .0001
Codman Hakim programmable valve	Device # 1 200 mm H_2_	20 10 6 4	5.77 (± 0.03) 3.81 (± 0.03) 2.08 (± 0.01) 1.71 (± 0.01)	2.16 2.26 1.03 0.86	< .0001 < .0001 < .001 < .001
	Device # 2 40 mm H_2_O	20 10 6 4	1.34 (± 0.02) 0.68 (± 0.05) 0.12 (± 0.01) 0.01 (± 0.01)	-2.27 -0.87 -0.93 -0.84	< .0001 < .001 < .001 < .001
	Device # 3 30 mm H_2_	20 10 6 4	2.67 (± 0.02) 0.39 (± 0.00) 0.00 (± 0.00) 0.00 (± 0.00)	-0.94 -1.16 -1.05 -0.85	< .001 < .001 < .001 < .001

Legend: no. = number, # = number, CSF = cerebrospinal fluid, DP = differential pressure, SD = standard deviation, mm = millimeter, cm = centimeter, H_2_O = water head

### Follow-up assessment

The mean follow-up time was 19.3 ± 3.8 months. In 6/8 (75.0%) patients clinical symptoms improved after valve replacement. All patients (100%) suffering from gait disturbances improved, as assessed by 10-m walking and 360°-turning performance (mean improvement: 26.7 ± 4.5% and 30.3 ± 4.8%, respectively). Cognitive deterioration and vertigo improved in 4/5 (80%) patients and urinary incontinence improved in 3/4 (75%). The preoperative intention tremor was no longer detectable postoperatively in 2/2 (100%) patients, as was the reduced vigilance status in 1/1 patient (100%). In average 1.5 ± 0.5 re-adjustments of opening pressure were required after valve replacement until symptoms improved. In 1/8 (12.5%) patient with a Miethke proGAV 2.0 valve, an additional implantation of an ASD was necessary after 13 months due to persisting symptoms of overdrainage after valve replacement. One patient died of metastatic cancer eleven weeks after revision of the valve, unrelated to surgery.

The suspected diagnosis of either over- or underdrainage correlated well with the measured flow rates in 7/8 (87.5%) of patients (Pearson correlation coefficient *r* = 0.71, 95% CI 0.42–0.88, *p* = 0.01). In 1/8 patient (12.5%) with a Codman Hakim programmable valve, the suspected diagnosis of overdrainage did not correlate with the decreased flow rates.

## Discussion

Taking into consideration that this is a very small study with a small number of valves tested and that its results must thus be interpreted with caution, one could speculate that VPS failure due to the valve itself might be underrepresented in the literature.

A mechanical malfunction of the shunt valve itself leading to an altered flow rate seems almost impossible to be detected by clinical and radiological diagnostic procedures. When revision of the entire shunt system is performed in the further course, shunt malfunction will of course be corrected, but the option of attributing the malfunction to the valve itself is precluded. In our study, all explanted shunt valves showed significant flow alterations at each DP in comparison to the corresponding new reference valve. Although postoperative in-vitro assessment located the underlying malfunction to the valve itself, the applied diagnostic procedures prior to revision surgery were not able to do so.

### Valve-dependent malfunction

Data on the underlying mechanical failure of a valve within a VPS is scarce. Aschoff et al. tested 36 constructions of 13 manufacturers in the “Heidelberger Valve Test Inventory” in 1990 and reported that only 28% of the new valves and 30% of the explanted valves met the manufacturer specifications whereas the majority of the valves showed serious deficits in terms of accuracy, long-term-stability and adequacy of flow-rates [4]. We are aware of the fact that the mentioned tests were performed in the 1980s and early 1990s. However, our results confirm significant flow alterations of the explanted valves, thus suggesting that long-term stability of shunt valves, even in the current generation, is still not reliable. From this point of view, our results do not corroborate the low incidence of valve-related complications leading to shunt failure and subsequent operative revision of previous studies [31, 41]. Reports on the valve itself as causative agent are elusive, often in the form of case reports [20, 32, 38]. Some larger series have shown an incidence of around 2% for shunt failure due to the valve itself, not including mere valve obstruction [19, 31, 41]. These described failures include, but are not limited to, valves set unintentionally to a non-desired opening pressure and valves with reprogramming failure. Furthermore, a buildup of tissue within the valves might explain the differences in flow rates compared to reference valves. Ludwig et al. reported on 18 of 19 explanted valves (silicone or titanium-based) in children, which were colonized with cellular tissue [22]. From a laboratory point of view, Chari et al. state that small particles (10 µm, mimicking red blood cells) in the reagent tend to increase shunts’ resistance or block them permanently. They further argue that large particles (25 µm) can block shunts, but commonly tend to open them permanently. However, they add restrictively that it is not clear whether microspheres reliably mimic the presence of particles in CSF [7]. Valves that are compromised this way may show partial obstruction only or even a higher flow-rate, resulting in a continued CSF-flow through the system. This is also the basis for the assumption that even with the help of contrast-enhanced shunt series, in addition to cranial imaging, the detection of incorrectly set valves with a wrong flow rate will not succeed. In this context, it has to be considered that the reliability of ventricular behavior on cranial CT or MRI scans used to exclude shunt malfunction in pediatric patients has been questioned [17]. This is largely consistent with our clinical experience in patients with long-term and normal pressure hydrocephalus, where clinical shunt malfunction is not always associated with changes in the ventricular system. In addition to a limited brain compliance, the cause may also lie in the fact that flow of CSF is not interrupted but continues at an unknown flow rate [20, 32, 33, 38]. Hassler et al. provide indications therefore, as they describe five cases of valve invagination in ventriculo-atrial shunting (Holter valve), where the atrial catheter was disconnected and the distal metal part of the valve had slipped into the silicone casing of the valve [15]. Only two of the five patients showed signs of raised intracranial pressure; the other three had some preservation of shunt function due to the formation of a fibrous sheath along the shunt [15]. Although the underlying cause of malfunction in this example is different from an error in the valve itself, it can still explain why corrupted valves with a preserved flow can escape detection in diagnostic imaging.

### Reasons for valve dependent malfunction

Meling et al. reported on valve-related failures of the gravity-dependent paedi-gav valve, representing the typical ball-cone-mechanism of the Miethke valves [26]. The explanted valves were tested preoperatively and found to be blocked in one, carry a constant resistance irrespective of valve orientation in five, carry a resistance other than stated in one, or had a valve resistance inadequate for the patient in two. In their paper on clinical experience with programmable Codman Hakim valves, Zemack et al. report on one valve with an opening pressure of 80 mmH2O instead of the 100 mmH_2_O at which it was set and another one with a fractured distal catheter [41]. Sato et al. describe a case of a Codman Hakim Programmable Valve (CHPV) with an opening pressure of 226 cm H_2_O instead of the adjusted a radiologically visible 60 mm H_2_O due to a head trauma [32]. The manufacturer’s examination of the extracted valve revealed a crack on the surface of the hard plastic housing covering the valve chamber, which appeared to be suggestive of some type of blunt trauma. In addition, the flat spring that transmitted resistance from the pressure control cam to the valve ball was deformed in such a manner that it caused excessive pressure against the valve ball. Rohde et a.l reported on 60 children with CHPV and found that three of their 31 complications were valve-related, with one malfunction, one obstruction, and one spontaneous change of the selected pressure setting and failure of the readjustment mechanism [31]. Mangano et al. reported on valve malfunction of programmable valves (CHPV; Medtronic Strata valve) in a pediatric population, where malfunction was defined as failure of the valve to open or close at an expected pressure. They found that a programmable valve itself failed in nine patients (11.1%/year of followup), necessitating shunt revision [23]. Lee et al. and Watanabe et al. reported on a case of overdrainage caused by detachment of the pressure control cam in a CHPV, respectively [20, 38]. In summary, the development of a valve malfunction seems to be primarily due to a mechanical impairment of the valve mechanics, some of which are quite complex. The vulnerability of these internal valve mechanics, oftentimes under long-term loading, can lead to undesirable changes in the flow rate in terms of under- or overdrainage, which remain undetected by the diagnostic options available.

### Past and future

Failure of CSF shunt of up to 50% at two years after implantation remains a persistent problem [9, 41]. Shunt obstruction and infection remain the predominant causes, whereas the valve itself is reported to be the site or cause of malfunction in the range of 2 to 3% [9, 41]. Although the exact reasons remain unclear, our study supports reduced long-term-stability followed by clinically relevant flow alterations even in the current generation of different types of shunt valves. If patients with implanted VPS present with disputable and less specific symptoms, unchanged ventricles in imaging and without diagnostics showing an adequate correlate, valve malfunction should be considered. Recently, Dias et al. reported on a software based shunt infusion study (SIS) with promising results, as it was deemed possible to prove or rule out VPS malfunction without the necessity of radiation exposure or surgery [8]. Although it does not allow for differentiation between valve obstruction and distal catheter obstruction, it would be of great clinical interest, whether shunt valves with defined characteristics (obstructive equals ICP plateau > 5 mmHg and borderline equals ICP plateau ≤ 5 mmHg above critical shunt pressure) exhibit correlating flow patterns in an in-vitro-setup like ours. This could be the subject of further studies. Furthermore, test series over a long time-period should be established to consider mechanical, chemical or iatrogenic influences on shunt valves to get closer to the cause of valve malfunction.

## Limitations

The major limitation of the study is the small number of shunts investigated. Thus, this study must be considered only as a pilot study and therefore its results may not be generalizable. Another limitation is the relatively short measuring time per valve (three times one minute). This might be considered insufficient to detect flow changes over time. However, we would also like to refer to previous studies of ours where we proceeded in the same way and the measurements of the valves consistently showed plausible values [10, 11].

In addition, the explanted valves were compared to only one respective reference valve. Considering that significant variations between the actual performance of valves and the manufacturer’s own specifications are described in the literature, this approach might be deemed inadequate [1–3, 34]. However, the flow rates of the reference valves were not considered absolute but in relation to the flow rates of the respective explanted valve. As all patients suffered from clinical symptoms of whether over- or underdrainage without evidence of other reasons of shunt failure, we assume that the displayed flow alterations are correct. Furthermore, besides the gravity-controlled, non-adjustable Miethke Shuntsassistant, only programmable valves (PV) were investigated in our study. This leads directly to the question of whether it would not be useful to compare the recently used non-programmable valves (NPV) and PVs in terms of their mechanical failure rate. Previous studies comparing the outcome of PVs with NPVs in hydrocephalic patients rarely make reference to an underlying mechanical failure of the valve, and when they do, they do so without sufficient workup [6, 16, 18, 21, 23–25, 28–30, 40]. Therefore, further studies comparing mechanical failure in PVs and NPVs are warranted.

As the aim of our study was purely to investigate whether a particular clinical and radiological pattern advocates revision of a suspected dysfunctional shunt valve, we naturally make no claim that this approach constitutes the only reasonable tool or technique to diagnose CSF shunt malfunction. A very sophisticated approach to detect underdraining shunts is provided by constant rate infusion studies. Unfortunately, CSF infusion studies are not routinely performed in our clinic, so this sophisticated test procedure unfortunately was not part of our armamentarium. Petrella et al. could show in their study that shunted patients in the underdraining group had higher baseline and plateau CSF pressures, higher resistance to CSF outflow and higher levels of baseline pulse amplitude waveform [27]. Reports of intraoperative shunt patency were available in 21 patients with suspected shunt blockage who subsequently underwent surgical revision of the shunt. Shunt blockade was confirmed intraoperatively in 19 cases. Weerakkody et al. used the computerized infusion test as a modification of the traditional constant rate infusion test and were even able to assess the difficult clinical problem of overdrainage in shunted patients [39].

## Conclusion

In all patients with suspected valve malfunction, our in-vitro characterization showed significant alterations of flow rates, suggesting a valve malfunction in terms of flow characteristics that differ from the manufacturer’s suggestions, which could not be objectified by the diagnostic tools used in the clinical routine. Although in these cases, VPS are still functioning, the altered flow rates might have a significant impact in patients where the amount of CSF diversion is critical. If shunt malfunction is suspected without evidence in diagnostics, and valve adjustments fail to improve the clinical situation, malfunction of the valve itself should be considered. Taking into account the limited number of the measured valves, this study must be considered merely a pilot study and therefore its results may not be generalizable. Further studies are required to confirm these findings.

## Data Availability

Not applicable.
